# Markov Models of Amino Acid Substitution to Study Proteins with Intrinsically Disordered Regions

**DOI:** 10.1371/journal.pone.0020488

**Published:** 2011-05-27

**Authors:** Adam M. Szalkowski, Maria Anisimova

**Affiliations:** 1 Swiss Institute of Bioinformatics, Lausanne, Switzerland; 2 Computational Biochemistry Research Group, Computer Science Department, ETH Zurich, Zurich, Switzerland; University of South Florida College of Medicine, United States of America

## Abstract

**Background:**

Intrinsically disordered proteins (IDPs) or proteins with disordered regions (IDRs) do not have a well-defined tertiary structure, but perform a multitude of functions, often relying on their native disorder to achieve the binding flexibility through changing to alternative conformations. Intrinsic disorder is frequently found in all three kingdoms of life, and may occur in short stretches or span whole proteins. To date most studies contrasting the differences between ordered and disordered proteins focused on simple summary statistics. Here, we propose an evolutionary approach to study IDPs, and contrast patterns specific to ordered protein regions and the corresponding IDRs.

**Results:**

Two empirical Markov models of amino acid substitutions were estimated, based on a large set of multiple sequence alignments with experimentally verified annotations of disordered regions from the DisProt database of IDPs. We applied new methods to detect differences in Markovian evolution and evolutionary rates between IDRs and the corresponding ordered protein regions. Further, we investigated the distribution of IDPs among functional categories, biochemical pathways and their preponderance to contain tandem repeats.

**Conclusions:**

We find significant differences in the evolution between ordered and disordered regions of proteins. Most importantly we find that disorder promoting amino acids are more conserved in IDRs, indicating that in some cases not only amino acid composition but the specific sequence is important for function. This conjecture is also reinforced by the observation that for 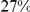 of our data set IDRs evolve more slowly than the ordered parts of the proteins, while we still support the common view that IDRs in general evolve more quickly. The improvement in model fit indicates a possible improvement for various types of analyses e.g. *de novo* disorder prediction using a phylogenetic Hidden Markov Model based on our matrices showed a performance similar to other disorder predictors.

## Introduction

Contrary to the traditional sequence-structure-function paradigm, the function of a protein is not determined solely by its stable 3D structure. Today it is known that naturally unfolded or so called intrinsically disordered proteins (IDPs) fulfill a multitude of functions, such as signaling and regulation. While some proteins are completely unstructured, others may contain only short disordered regions. Current estimates suggest that more than 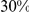 of eukaryotic proteins contain long intrinsically disordered regions (IDRs), but IDRs are also frequently found in prokaryotes [1]. According to estimates from Ward *et al.* [2], on average 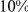 of proteins are fully unstructured, while half of all proteins contain at least one long IDR. IDPs (or proteins with IDRs) often depend on structure instability for their function [3]–[5]. The absence of a stable 3D or secondary structure makes IDPs more flexible when binding and forming protein complexes, providing important advantages over ordered proteins [6]. Compared to ordered proteins, IDPs often participate in molecular recognition, signaling processes, cell-cycle regulation and modulating gene expression or chaperone activity [7]. Due to their flexibility, IDPs are more resistant to perturbations in the molecular interactions environment and tend to act as hubs in molecular interaction networks [8]. Proteins with IDRs are increasingly associated with diseases such as cancer and neurodegeneration [7]. For example, the CREB transcription factor is crucial in neuronal plasticity and long-term memory formation in the brain; malfunctions of CREB may contribute to the development of Huntington's disease and some types of cancers. Other famous examples include prion protein and tumor suppressor proteins p53 and BRCA1 [7], [9].

To date most studies contrasting the differences between ordered and disordered proteins focused on simple summary statistics, such as sequence complexity and amino acid composition [10], [11]. For example, regions of low sequence complexity are likely to be disordered [10]. IDRs usually have few large hydrophobic residues but favor polar and charged amino acids. Such sequence composition properties are often used by computational methods of disorder prediction (see [12]).

Brown *et al.* [11] estimated separate Markov amino acid substitution models for ordered and (wholly) intrinsically disordered proteins at three levels of sequence similarity. These models were used to compare amino acid frequencies and average rates of evolution. The authors concluded disordered proteins having a generally higher rate of evolution than ordered. Midic *et al.* [13] published a scoring matrix for the alignment of protein sequences with disordered regions. This study also confirms a higher rate of evolution in IDRs and shows differences in amino acid substitution patterns between ordered and disordered parts of proteins.

Here, we take an evolutionary approach and study multiple sequence alignments of homologous proteins with IDRs using Markov amino acid substitution models in the maximum likelihood (ML) framework. Based on a large set of homologous groups with experimentally annotated IDRs, we estimate two empirical amino acid substitution models, each describing the evolution either in ordered or disordered regions. An expectation-maximization (EM) algorithm is used to obtain ML estimates of model parameters [14]. A new method is suggested to evaluate whether components of two inferred substitution models are significantly different. This test shows that our models are indeed significantly different and capture the essential features of ordered and disordered regions. As using the new model with *a priori* known IDRs significantly improves the fit to data, the new model may be recommended for other downstream evolutionary analyses. For example, using the new two component order-disorder model we define a phylogenetic Hidden Markov Model (phylo-HMM) and apply it as a *de novo* predictor of intrinsic disorder in multiple sequence alignments of homologous proteins. Our predictor demonstrates the potential of achieving a competitive accuracy-power balance compared to other disorder prediction methods.

Further, the estimated empirical models were used to contrast patterns and rates of the evolution in disordered and the corresponding ordered protein regions. It is typically thought that disordered regions are in general faster evolving than structured regions of the same protein. While we confirm that IDRs tend to have a higher rate of evolution compared to the rest of the protein, we find a significant number of protein groups where the reverse is the case. We present examples of proteins where the evolutionary rates at IDRs are significantly slower (or faster) compared to the corresponding ordered regions. Finally, we discuss other properties of IDPs such as their distribution among functional categories and biochemical pathways and their preponderance to contain repeats in tandem (another important property correlating with enhanced protein binding [15]).

## Materials and Methods

### Assembling homologous protein groups with IDRs

Previous analyses [16] relied on the computational prediction of intrinsic disorder [13], [17]–[21]. Here, we decided not to use computational prediction methods. One one hand this drastically reduces the amount of data available for model estimation. On the other hand we avoid introducing unforeseeable biases due to prediction inaccuracies.

Instead our analyses were based on the DisProt database [22] as a starting point of data acquisition. This database comprises about 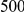 proteins annotated with a total of about 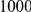 experimentally verified intrinsically disordered regions.

DisProt was scanned for the presence of homologous proteins using the BLASTCLUST program, which finds pairs of sequences with statistically significant matches (using the BLAST algorithm) and groups them based on single-linkage clustering. This program is part of the BLAST suite [23]. We found that the DisProt database contains very few homologs. Consequently, we expanded the set of IDPs through further searches for homologous proteins in SwissProt and Pfam-based PANDIT databases. The similarity threshold was set to be sufficiently stringent to assume structural homology so that disorder annotations could be propagated to all homologous positions. A more detailed description of this procedure is provided below.

### PANDIT data set

Each DisProt sequence entry was mapped to a representative homologous group in the PANDIT database [24] based on pairwise local alignment [25] score with BLOSUM62 [26]. The score threshold was set to 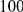 which corresponds to an E-value of 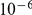. PANDIT consists of Pfam protein families [27] together with multiple sequence alignments and inferred phylogenetic trees based on protein-coding DNA and amino acid sequences.

When multiple DisProt sequences mapped onto a single homologous group in PANDIT, the group was successively bisected by its longest branch until the mapping became injective, so that there was only one disorder annotation per group. Groups with no mapping or with 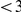 taxa were discarded. The corresponding multiple sequence alignments were restricted to the homologous sites as determined by the pairwise alignments to the reference sequence from DisProt. To avoid noise in the matrix estimation, distant sequences were filtered out based on the alignment score. The final set contained 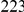 homologous groups with a total of 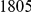 sequences with 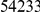 disordered and 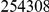 ordered residues.

The PANDIT data comprises a set of reliable alignments but due to its limited size it imposes considerable uncertainty on model estimation. Thus we use this data set mainly for verification of our results and for functional analyses.

### SwissProt data set

To improve the reliability of our model estimation, we constructed a second larger data set of homologous protein groups with IDRs based on the SwissProt database [28]. For each DisProt entry an initial homologous group from SwissProt sequences was built from pairwise alignments. Multiple sequence alignments were constructed from pairwise homologies and trimmed to sites present in the reference sequence. Further, the groups were refined by removing distant sequences so that each sequence had a distance 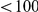 PAM to the reference sequence. The resulting data set included 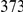 homologous protein groups with a total of 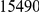 sequences with 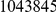 disordered and 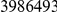 ordered residues and was used as the main source for the estimation of our Markov model of evolution. To overcome potential biases due to errors in the group-wise multiple sequence alignments and estimated phylogenies, we compare the separate model estimates for both data sets.

### The estimation of Markov amino acid substitution models

The evolution of amino acids was described by a Markov process with the generator matrix 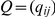 defining the instantaneous rates of changes from amino acid 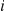 to 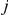. As usual, the substitution process was assumed to be reversible so that 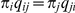, where 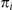 are the *equilibrium amino-acid frequencies*. For a reversible process the instantaneous rates of change from 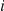 to 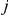 can be expressed as 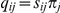, a product of equilibrium (or stationary) amino acid frequency 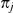 and the exchangeability 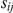 between residues 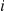 and 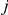. We further refer to the matrix 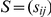 as the amino acid exchangeability matrix. For a multiple sequence alignment the substitution process flows along a phylogeny relating the sequences in a sample. The transition probability matrix over time 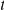 is computed as 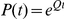. On this basis a likelihood function can be constructed for each site for a given tree. The total likelihood of the alignment is calculated as a product of site likelihoods based on the site-independence assumption (for computational reasons).

We estimated separate amino acid substitution models for ordered and disordered regions, each described with instantaneous substitution matrices D and O, respectively. Overall, the mixed *DO* model describes the evolution of *a priori* annotated IDRs using matrix *D*, while structured regions are described using matrix *O*.

Model parameters were estimated using an EM algorithm [14] on our two assembled training sets. The EM approach finds the ML estimates of substitution model parameters, with the substitution histories and counts being unobserved latent variables. The EM iteratively estimates parameters and latent variables in an alternating manner until convergence. Each model (both for ordered and disordered regions) required estimating 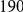 exchangeability and 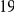 amino acid frequency parameters.

For the training set based on PANDIT groups we used phylogenies provided by the PANDIT database. For the SwissProt data set phylogenies were built using PhyML3.0 [29] with 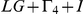 [30], [31], thereby estimating evolutionary rates per site.

We followed the procedure described by Le *et al.* [30] and separated the alignment columns by their most likely rate class, as estimated by PhyML, to normalize for among-site heterogeneity of evolutionary rates.

### Evaluating the significance of differences between models estimated for ordered and disordered regions

The significance of differences in estimated amino acid frequencies was evaluated using two likelihood-ratio tests on the estimated amino acid counts computed by XRate [14]: Pearson's 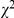-test and the G-test. Both tests compare the null hypothesis that the two count vectors arose from a common distribution against the alternative hypothesis where each vector originates from a distinct distribution. Similarly, exchangeability rates were compared using the estimated substitution counts.

**Figure 1:**
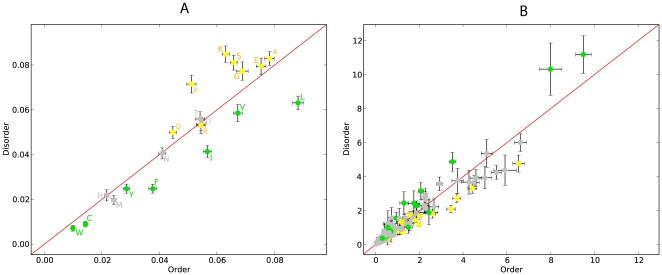
Scatter plot for amino acid frequencies (A) and exchangeablilities (B) in SwissProt data set. Error bars are 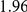 standard deviations. Order promoting amino acids are green, disorder promoting ones yellow. Exchangeabilities between order and disorder promoting residues are gray.

In addition to Pearson's 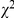 and the G tests, confidence intervals for model estimates were computed by a bootstrapping technique. For each homologous group replicate data sets were generated by bootstrap on alignment columns and by jackknife on rows. For each replicate, substitution models for ordered and disordered regions were re-estimated using the EM-based procedure identical to that applied to the original data. The resulting distributions of model estimates were used to estimate empirical variances for exchangeabilities and amino acid frequencies (Figure 1).

In particular, we investigated whether the IDRs may be characterized only by the bias in amino acid composition, or if a bias in exchangeability between different classes of amino acids (order and disorder promoting) may also be observed. To achieve this we computed the substitution rates between order and disorder promoting residues for ordered and disordered regions separately:
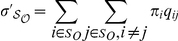


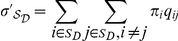
where 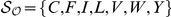 and 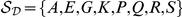 are the sets of order promoting and disorder promoting amino acids, respectively. In order to compare these terms between ordered and disordered regions we normalized the terms by frequencies of occurrences of amino acids in sets 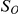 and 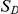:
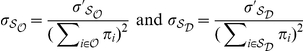
which rendered these terms independent of the target and source amino acid frequencies. To detect bias in exchangeabilities with regard to order and disorder promoting residues, we compared the ratios 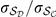 between ordered and disordered regions.

### Comparison of evolutionary rates in ordered and disordered regions

To compare average rates of evolution we computed the group-wise total tree lengths (sum of branch lengths) for the SwissProt data set from pairwise distances and least-squares distance trees estimated with Darwin [32], because for a given set of taxa tree lengths are expected to be proportional to the average rates of evolution. We will refer to the estimated evolutionary rates as 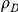 for IDRs and 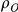 for ordered regions.

The ordered and disordered portions of multiple alignments were bootstrapped separately, and the significance was computed with the Mann-Whitney-U-Test.

### Prediction of IDRs using phylogenetic Hidden Markov Models (phylo-HMMs)

The estimated empirical models for ordered and disordered regions may be used to define a phylo-HMM for predicting IDRs. We applied XRate [14] in annotation mode to obtain a prediction of order/disorder for each alignment column in the testing set compiled from PANDIT. This was done using the model estimates for order and disorder trained on either the PANDIT or the SwissProt sets. The HMM consists of 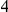 hidden states: start, end, and states for emitting ordered and disordered alignment columns. The emission probabilities are defined by the estimated evolutionary model and the transition probabilities were trained simultaneously from data. To correct for the differences in group size we divided the error statistics by the corresponding number of sequences in the homologous group.

The quality of prediction of intrinsic disorder for our phylo-HMM was compared with the quality of two sequence-based disorder predictors: VSL2 [20] and iupred [21]. VSL2 was used with two different parameter sets. One version of VSL2 uses auxiliary information from PSI-Blast PSSM and PSI-Pred secondary structure prediction, while the “fast” version is executed without this additional data. Iupred was used with its “long” and “short” presets.

## Results

The new *DO* model requires twice as many parameters to be estimated from data compared to a standard empirical amino acid model that does not distinguish between order and disorder. Despite this, the model significantly improved the model fit to data with *a priori* annotated IDRs. For example, for the SwissProt data set the AIC decreased by 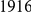 (with an increase in log-likelihood of 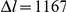). Consequently, we used the DO model to analyze differences between ordered and disordered regions in terms of amino acid composition and exchangeabilities, evolutionary rates, and content of tandem repeats. We also tested whether the two components of *DO* may be used for disorder prediction from multiple alignments of homologous protein sequences.

### Comparison of model estimates

We compared the ML estimates for the disorder and order components of the *DO* model in the PANDIT and SwissProt data sets. The majority of the ML estimates were similar between the two data sets. Only for a few amino acids the estimated equilibrium frequencies differed significantly between the data sets, based on variances estimated with bootstrap and jackknife resampling (Supplementary Figure S1). This may reflect a heterogeneity of gene and lineage composition. On the other hand, no significant differences were observed between exchangeability estimates in the two training sets. Such stability of our estimates is reassuring.

The uncertainty in model estimates for the SwissProt training set was lower compared to that for the PANDIT set. Moreover, we observed a lower variance in the estimates for the ordered regions compared to the disordered model. This may be explained by the amount of data available for each estimation, since the SwissProt set is larger than the PANDIT set, and since we have more residues in ordered regions compared with IDRs. Consistent with this explanation, we observe high variance in exchangeabilities between rare amino acids.

**Figure 2:**
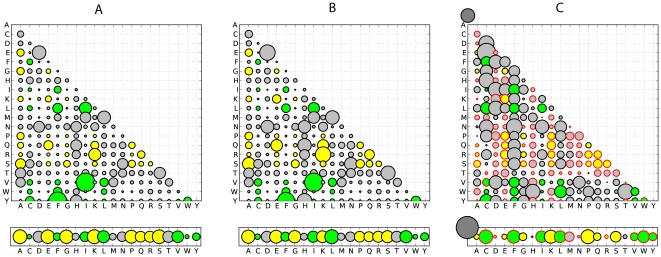
Amino acid exchangeability matrices and amino acid frequencies for ordered and disordered regions derived from the SwissProt data set: here, the area of each bubble represents the rate of a substitution or the amino acid frequency. (A) Model estimates for IDRs. (B) Model estimates for ordered regions. (C) Relative difference (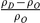) between the corresponding values for disordered and ordered models (plots A and B). 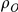 and 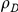 stand for the relative evolutionary rates in ordered and disordered regions, respectively. Order promoting amino acids are green, disorder promoting ones yellow. Exchangeabilities between order and disorder promoting residues are gray. Bubbles with red border correspond to negative values, i.e. have a lower frequency in IDRs.

Next, we contrasted model estimates for ordered and disordered regions in the SwissProt data set. The estimates of model parameters for IDRs are shown in figure 2 and can be downloaded as supplementary datasets S3 and S4 in a format compatible with PAML [33]. The amino acid equilibrium frequencies and exchangeabilities are displayed and compared separately. The components of *O* and *D* matrices for ordered and disordered models were found to be significantly different based on Pearson's 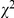-test and the G-test (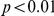 for both tests) applied to estimated substitution counts.

Based on the estimates of amino acid frequencies for ordered and disordered regions (Figure 2), we observed that order-promoting amino acids I, L, V (large and hydrophobic) and W, Y, F (aromatic) appeared in IDRs at a lower frequency. In addition, IDRs contained a low frequency of the non-polar amino acid C. On the other hand, IDRs contained high frequencies of disorder-promoting amino acids: positively charged R and K, polar E and Q, and small A, G, S and P. Our estimates of amino acid frequencies were largely in agreement with other empirical observations [10], [11]. Our observations held for both data sets with only minor differences.

We clearly observed that IDRs are enriched with disorder-promoting amino acids while ordered regions are enriched with order-promoting amino acids. Further, significant differences in the amino acid exchangeability patterns between the models inferred for ordered and disordered regions were found (Figure 1).

In IDRs we observed relatively fewer substitutions between disorder promoting residues compared to ordered regions (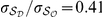 in IDRs and 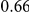 in ordered regions). In addition, in IDRs the exchangeability rates are higher between order-promoting residues, whereas in the ordered regions the exchangeability rates tend to be higher between disorder-promoting residues and between residues from the two classes (order or disorder promoting). Thus, it may be concluded that IDRs are characterized not only by the compositional bias but also by exchangeability biases between the classes of order and disorder-promoting residues.

### Performance of HMMs for *de novo* disorder prediction

Using a test data set compiled from PANDIT, we compared the performance of our phylo-HMM based disorder predictor with two well established sequence-based predictors. Table 1 summarizes the numbers of correctly and incorrectly annotated sites with different methods tested. In our tests, VSL2 exposed the best performance in marking sites as disordered, while Iupred was too conservative, annotating too many sites as ordered. According to precision and recall values (Table 1), our phylo-HMMs outperformed the simple sequence based Hidden Markov Models based only on amino acid frequencies and yield results comparable to iupred and VSL2. It should be noted that VSL2 and iupred preformed similar or even better on the test set compared to predictions on DISPROT (results not shown).

**Table 1:** Comparison of disorder prediction.

Tool	TP	TN	FP	FN	accuracy	recall
VSL2 fast	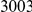	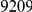	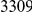	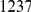	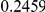	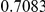
VSL2	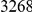	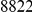	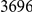	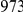	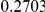	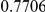
iupred long	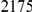	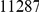	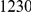	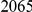	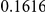	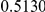
iupred short	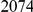	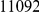	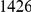	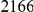	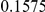	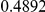
phyHMM SwissProt	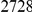	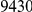	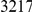	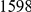	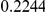	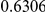
phyHMM PANDIT	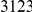	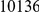	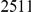	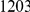	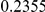	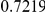
HMM SwissProt	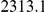	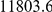	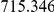	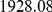	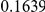	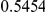
HMM PANDIT	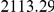	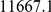	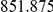	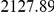	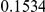	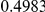

Comparison of phylo-HMM based disorder prediction using the models estimated from the PANDIT or the SwissProt data set with other sequence based predictors. Shown are true positives (TP), true negatives (TN), false positives (FP), false negatives (FN) for different parameter configurations of each method.

### Comparison of evolutionary rates in ordered and disordered protein regions

It is typically thought that IDRs evolve at a higher rate compared to proteins with stable 3D structure [11], [34]. Here, we tested this consensus view using the rate estimates from our inferred models.

For about 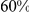 of the PANDIT data set the evolutionary rates were significantly higher in IDRs compared to rates in ordered regions (Table 2). For example, the tumor suppressor protein p53 (PF00870) was found to evolve significantly faster in its IDR (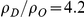). Another example from this class is discussed below in more detail. However, for 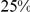 of our homologous groups the estimated rate of evolution in IDRs was significantly lower than in respective ordered regions (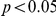). The full results are available in supplementary dataset S1 (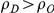) and S2 (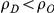). This may indicate that the contribution of IDRs to the overall function of the protein may vary significantly, and which is confounded with a multitude of other factors including the properties of the primary sequence.

**Table 2:** Comparison of evolutionary rates.

	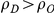	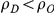	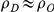
LG	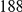	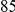	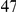
DO	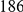	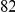	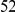

Comparison of evolutionary rates between ordered and disordered columns in the SwissProt data set. Each cell contains the number of homologous groups which pass a test of significance at 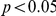 or the number of those with indistinguishable rates.

Further we explored the distribution of functional categories among groups with either significantly higher or lower evolutionary rates between ordered and disordered residues. For this task we used the PANDIT dataset since the information on functional categories (GO [35] terms) and biochemical pathways (KEGG [36]) was already available from PanditPlus [37]. For each protein group we parsed GO terms from the highest hierarchical level down to collect all relevant ancestral terms. The class with higher rate in IDRs (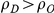) was enriched with proteins from the functional categories *‘nucleotide binding’* (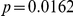, 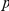-values before multiple testing correction), and especially *‘adenyl nucleotide binding’* (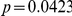) and *‘ATP binding’* (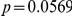).

In the other class with 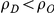 the cellular component *‘membrane part’* (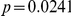) and the biological process *‘regulation of nucleobase, nucleoside, nucleotide and nucleic acid metabolic process’* (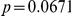) were overrepresented. Due to the small number of homologous groups we had, these were the only terms close to a significant level. However, functional categories such as binding and regulation were reported to be enriched in other studies, which used disorder predictors for such analyses [38], [39].

For KEGG pathways we found that proteins with less conserved IDRs (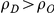) tend to be involved in *‘proteasome’* (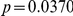),‘*apoptosis*’ (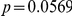) and *‘colorectal cancer’* (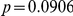) pathways. Proteins with conserved IDRs (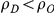) were found to be highly significantly overrepresented in *‘tyrosine metabolism’* (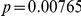) as well as *‘pyruvate metabolism’* (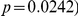, *‘valine, leucine and isoleucine degradation* (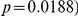, *‘urea cycle and metabolism of amino groups’* (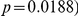, *‘1- and 2-methylnaphthalene degradation’* (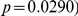, *‘fatty acid biosynthesis’* (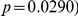, and *‘3-chloroacrylic acid degradation’* (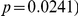 pathways. Interestingly, according to KEGG most of these pathways fall into the same larger category or/and are related.

Note that the estimates of tree lengths were robust to the model choice (estimates for new model *DO* and LG differed by only 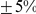), and thus had little influence on our conclusions regarding the comparisons of the evolutionary rates 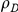 and 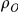 (Table 3).

**Table 3:** Overlaps between rate estimate classes.

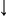 LG/DO 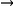	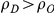	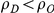	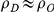
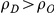	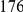	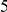	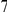
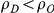	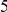	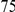	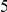
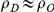	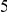	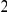	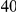

Overlaps between different rate estimate classes based on the LG and the *DO* models. Especially the overlaps between the opposing classes are within the targeted level of confidence (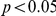). The estimates based on the LG and DO models are not significantly different. Thus, using a single model for rate estimation is considered sufficient.

### Relationship between intrinsic disorder and protein repeats in tandem

It has been suggested that about 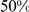 of the protein regions with tandem repeats may be intrinsically disordered [40]–[42], implying a higher incidence of IDRs in proteins with tandem repeats compared to their average frequency among all proteins. Here, we examined whether the reverse observation may be made, i.e. proteins with IDRs are more likely to contain tandem repeats. To assess whether IDPs are enriched with tandem repeats, we examined the frequency of tandem repeats in our homologous groups. For each group in our SwissProt data set and for each DisProt sequence, protein repeats were detected using a recent algorithm based on a k-means clustering approach [43]. We found that 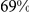 (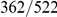) of the sequences in DisProt contained predicted repeats and that 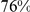 (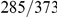) of our SwissProt groups contained at least one sequence with tandem repeats. This is significantly higher than what is typically observed among all proteins (reportedly 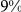 in SwissProt and 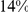 in a GenBank-based protein census [44]).

This analysis demonstrated that tandem repeats tend to occur more frequently in intrinsically disordered regions (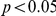; Table 4). Furthermore, in our data proteins with tandem repeats tended to have higher rates of evolution in IDRs (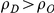) more frequently compared to proteins without tandem repeats (Table 4).

**Table 4:** Intrinsic disorder and tandem repeats.

	# residues in order	# residues in disorder	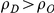	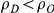	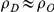
TR	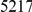	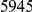	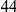	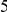	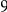
noTR	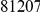	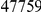	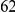	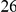	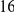

The first 2 columns contain numbers of ordered or disordered characters in DisProt which are predicted to be inside or outside of tandem repeats. Tandem repeats are significantly more frequent in disordered regions (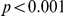).

The last 3 columns represent a comparison of evolutionary rates between ordered and disordered columns in the SwissProt data set restricted to groups with or without tandem repeats, respectively. Each cell contains the number of homologous groups which pass a test of significance at 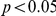 or the number of those with indistinguishable rates.

### Examples of proteins with IDRs

#### 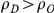 in mouse SOCS3

**Figure 3:**
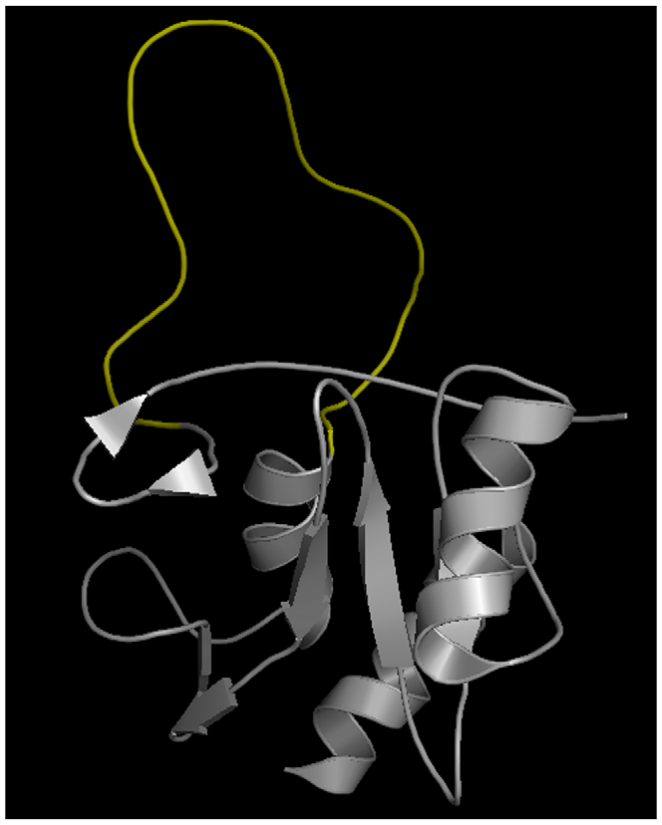
In murine SOCS3 the IDR (yellow) between the SH2 domain and the SOCS box is little conserved. It presumably just has an effect in the degradation of the protein. This structure is available as PDB identifier 2BBU.

Significantly higher rate of evolution in the IDR compared to the ordered portion of the protein was found in the homologous protein group constructed for the DISPROT sequence DP00446. This protein is a suppressor of cytokine signaling (SOCS3) in mouse. The IDR between the SH2 domain and the C-terminal SOCS box (Figure 3) is believed to be a PEST-like sequence and is not required for primary function (phosphothyrosine binding) [45]. Instead it is likely to have an enhancing effect in protein degradation.

SOCS3 is involved in the following GO biological processes: ‘*branching involved in embryonic placenta morphogenesis*’, ‘*negative regulation of insulin receptor signaling pathway*’, ‘*negative regulation of signal transduction*’, ‘*placenta blood vessel development*’, ‘*positive regulation of cell differentiation*’, ‘*regulation of growth*’, ‘*regulation of protein phosphorylation*’, ‘*spongiotrophoblast differentiation*’, ‘*trophoblast giant cell differentiation*’. Further it is annotated with the molecular function ‘*protein binding*’. The protein is part of the following KEGG pathways: ‘*Ubiquitin mediated proteolysis*’, ‘*Osteoclast differentiation*’, ‘*Jak-STAT signaling pathway*’, ‘*Insulin signaling pathway*’, ‘*Adipocytokine signaling pathway*’, ‘*Type II diabetes mellitus*’, ‘*Hepatitis C*’.

We conducted a more thorough analysis of this protein and assembled a superset of this homologous group from the OMA project [46]. By doing so we obtained a group of 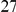 sequences and a total alignment length of 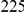 amino acids with 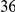 disordered columns. Babon *et al.* [45] note that the IDR of this protein is highly conserved in mammals. Despite this, our analysis confirmed a significantly higher substitution rate in the IDR compared to the rest of the protein. For this data set the estimated average tree length measured in expected substitutions per site was 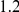 in ordered regions but 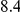 for the IDR, with highly significant Mann-Whitney-U-Test. Further, protein-coding DNA sequences were analyzed using codon models with variable selection pressure over sites (models M0, M1, M2, M3, M7 and M7 in PAML [33]). No positive selection was detected on this protein, but the purifying selection pressure was less stringent in the IDR compared to the ordered part of the protein - the trend consistent with our observation of 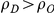.

#### 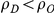 in rat GNMP

**Figure 4:**
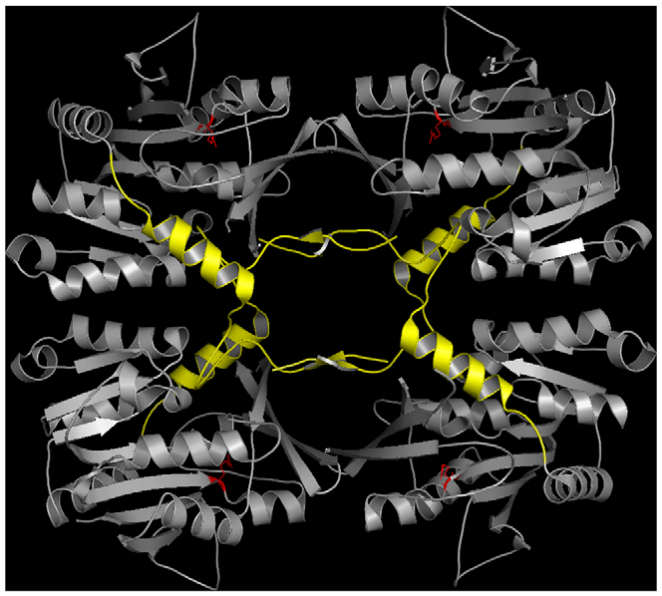
In rat GNMP the N-terminal IDRs (yellow) are strongly conserved. They give access to the active sites (red) in the presence of AdoHcy. This structure is available as PDB identifier 2IDJ.

The Glycine N-methyltransferase (GNMP) is an example of a protein where the rate of evolution is significantly lower in the IDR compared with the ordered regions of the protein - contrary to the predominant view. This protein creates a tetrameric complex shaping a molecular basket. The 40 unstructured N-terminal residues of each subunit regulate access to the active site by filling the core of this basket (Figure 4). In presence of AdoHcy these IDRs unclog the core and give access to the active site [47].

GNMP is involved in the following GO biological processes: ‘*adenosylhomocysteine metabolic process*’, ‘*S-adenosylmethionine metabolic process*’, ‘*folic acid metabolic process*’, ‘*protein homotetramerization*’. Further it is annotated with the molecular functions ‘*folic acid binding*’, ‘*glycine N-methyltransferase activity*’, and ‘*glycine binding*’. The protein is part of the KEGG pathway ‘*Glycine, serine and threonine metabolism*’.

Similar to SOCS3, we expanded the original homologous group containing GNMP (around the DisProt sequence DP00031) with additional sequences from OMA. Thus this group was extended from 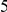 (originally) to 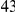 taxa and a total alignment length of 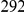 amino acids with 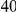 disordered columns.

The analysis of the extended dataset resulted in the estimated average tree length of 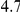 for the ordered regions versus 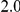 for the IDR, with highly significant Mann-Whitney-U-Test. The analyses with codon models (as for SOCS3) reported higher purifying selective pressure in the IDR. This again confirms our previous result suggesting that the IDR in GNMP protein is more conserved although it does not contain the active site.

## Discussion

Here we estimated an empirical Markov amino acid substitution model for IDRs and ordered regions of proteins, which provided a significant improvement in model fit to data (as measured by AIC). Based on the *a priori* annotated alignments, the mixed *DO* model succeeded at detecting several significant distinctions between evolutionary patterns in IDRs and the corresponding structured parts of the protein. First, the stationary amino acid distribution was found to be significantly skewed towards disorder promoting amino acids, which confirmed previous empirical observations [10], [11]. Moreover, the exchangeability rates in IDPs were also biased, with significantly higher rates between order promoting residues. At the same time, the exchangeability rates for other types of changes were lower compared to what was observed in ordered regions. Probably, in IDRs disorder promoting amino acids are under higher functional constraints than order promoting residues. As a result, the *DO* model may better reflect the biological reality for IDPs and therefore may improve the accuracy of inferences for various types of analyses, such as maximum likelihood phylogeny inference with mixture models [48], ancestral reconstruction, and sequence alignment. As an example, we used our model to construct a phylo-HMM to predict intrinsic disorder from a multiple sequence alignment of IDPs based on the difference in evolutionary patterns. The phylo-HMM based on the estimated models was shown to be competitive compared with other sequence-based predictors. Combining this approach with the use of summary statistics, such as energy calculations or the inclusion into a meta-predictor may improve the prediction even further. One limitation of our study was the relatively small sample of IDPs that are currently known to contain structural disorder based on experimental work. Recently however, the natively unfolded proteins have been under spotlight [49]. The increased attention to IDPs is likely to increase the amount of structural information available for this kind of proteins. Larger sets of homologous alignments of IDPs may be used in the future to re-estimate the two empirical components of our *DO* model. Such new estimates should be more accurate and have smaller variances. However, the composition of IDRs may depend on their relevance to the function of the protein and the specifics of performed function. Given sufficient data, different *DO* models may be estimated for different classes of IDPs, where IDRs play different functional roles. At the moment this is not foreseeable due to lack of both structural and functional data.

Our analyses suggest that for the majority (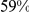) of IDPs the unstructured regions are indeed less conserved than the rest of the protein, as is typically thought. However, this is not a general rule and many exceptions exist. For 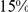 of IDPs the rates of evolution in IDRs and ordered regions were not significantly different. Moreover, a large proportion of IDPs in our set (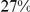) had higher conservation in their disordered parts, contradicting the common view (one such example, the GNMP protein, was presented above). Our functional enrichment analyses of this protein class showed that IDPs with 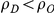 tend to be involved in pathways responsible for amino acid and carbohydrate metabolism or related. In particular, the amino acid metabolism function involved the metabolism of order promoting residues (but not disorder promoting) - namely Tyrosine, Valine, Leucine and Isoleucine. We hypothesize that this may be related to our observation of higher exchangeability rates between order-promoting residues in IDRs compared to ordered regions. Overall, IDPs with slower evolving IDRs (compared to their structured parts) seem to exhibit a preferential involvement in certain biochemical pathways. Indeed, proteins whose IDRs are directly involved in function, or are crucially important for function, may be expected to evolve more slowly due to additional functional constraints. In addition, IDRs abundantly found in alternatively spliced regions [50], [51] may evolve slower with respect to other regions due to additional constraints for functional proteins in different alternative frames.

A recent study [42] found that tandem protein repeats are enriched with IDRs. Here, we found that the reverse statement also may be made, i.e., proteins with IDRs are enriched in tandem repeats. So the presence of tandem repeats in a protein should have strong correlation with the presence of IDRs. This supports the theory that at least some of IDRs originate via repeat expansion [40]. This evolutionary mechanism provides a means of interactome scaling, where certain nodes in the interaction network increase their fitness by incorporating intrinsic disorder and repeats [9]. Sandhu [52] is also supportive of this view in his study of chromatin remodeling proteins that frequently contain IDRs. The IDRs resulting from repeat expansion may enable reversible binding to different interacting partners, which overall contributes to functional diversity and specialization of chromatin remodeling complexes. Moreover, Jorda *et al.* [42] found that the level of repeat perfection correlates with the amount of intrinsic disorder. If the repeat perfection is representative of recent evolutionary origin (rather than due to functional importance), then this finding is in a perfect agreement with the hypothesis that repeat expansion drives the origin of new IDRs. With time the repeat perfection should be decreased, especially that in our study we found that most IDPs with repeats evolve significantly faster in their IDRs compared to the structured regions. This may be also indicative that IDPs with tandem repeats fall into particular functional classes, a premise that should be studied when more structural and functional data (especially on IDPs) becomes available.

## Supporting Information

Figure S1
**Scatter plot of Pandit vs. SwissProt amino acid frequencies (A) and exchangeabilities (B) for the disordered model.** Error bars are 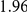 standard deviations. Order promoting amino acids are green, disorder promoting ones yellow. Exchangeabilities between order and disorder promoting residues are gray.(TIF)

Dataset S1 (CSV)

Dataset S2 (CSV)

Dataset S3 (QMAT)

Dataset S4 (QMAT)
