# Supplementary figures and images for: Markov Models of Amino Acid Substitution to Study Proteins with Intrinsically Disordered Regions
Source: PLoS One. 2011 May 27;6(5):e20488. doi: 10.1371/journal.pone.0020488 (PMC3103576; doi:10.1371/journal.pone.0020488)

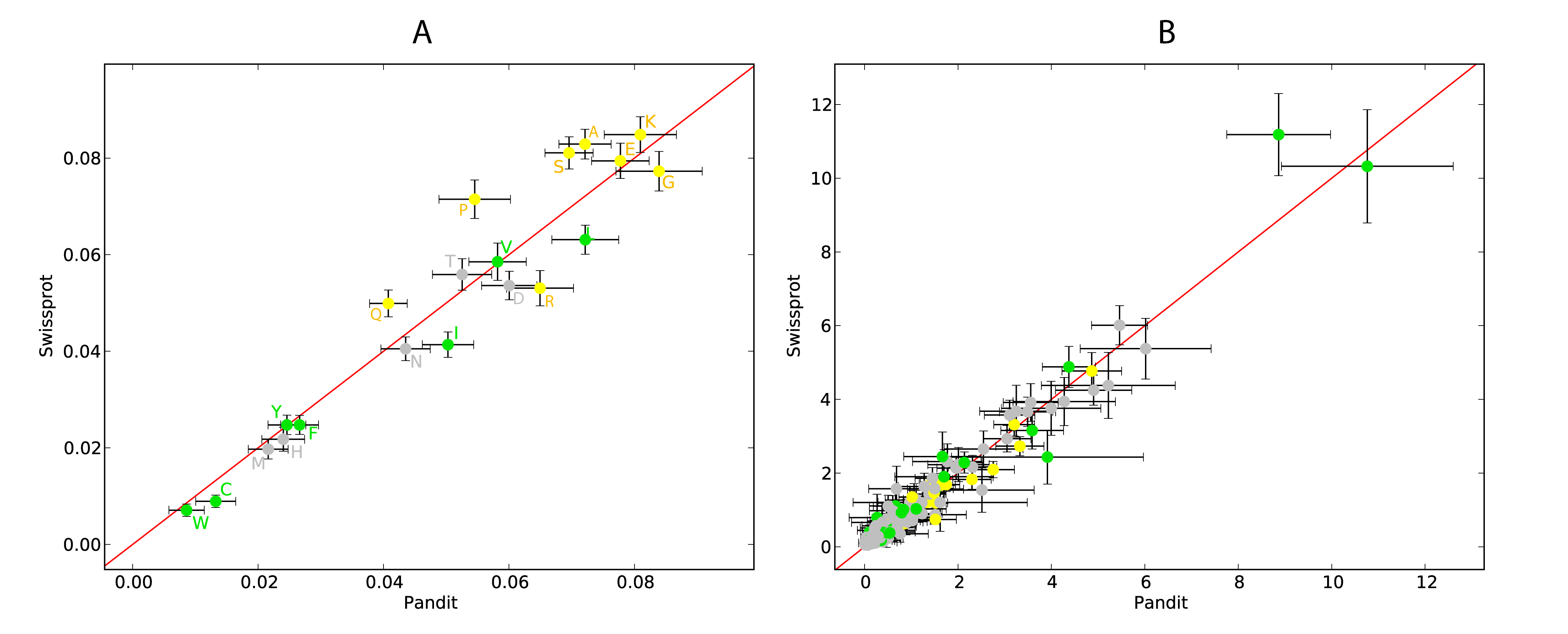

Supplement: Figure S1 — Scatter plot of Pandit vs. SwissProt amino acid frequencies (A) and exchangeabilities (B) for the disordered model. Error bars are standard deviations. Order promoting amino acids are green, disorder promoting ones yellow. Exchangeabilities between order and disorder promoting residues are gray. (TIF) [file pone.0020488.s001.tif]
